# Serial Analysis of the T-Cell Receptor β-Chain Repertoire in People Living With HIV Reveals Incomplete Recovery After Long-Term Antiretroviral Therapy

**DOI:** 10.3389/fimmu.2022.879190

**Published:** 2022-05-02

**Authors:** Andrea M. H. Towlerton, Shashidhar Ravishankar, David G. Coffey, Camille E. Puronen, Edus H. Warren

**Affiliations:** ^1^ Clinical Research Division, Fred Hutchinson Cancer Research Center, Seattle, WA, United States; ^2^ Division of Medical Oncology, University of Washington, Seattle, WA, United States; ^3^ Division of Hematology, University of Miami, Miami, FL, United States

**Keywords:** HIV, T-cell repertoire analysis, Antiretroviral therapy (ART), Immune restoration, TCR sequencing

## Abstract

Long-term antiretroviral therapy (ART) in people living with HIV (PLHIV) is associated with sustained increases in CD4^+^ T-cell count, but its effect on the peripheral blood T-cell repertoire has not been comprehensively evaluated. In this study, we performed serial profiling of the composition and diversity of the T-cell receptor β-chain (*TRB*) repertoire in 30 adults with HIV infection before and after the initiation of ART to define its long-term impact on the *TRB* repertoire. Serially acquired blood samples from 30 adults with HIV infection collected over a mean of 6 years (range, 1-12) years, with 1-4 samples collected before and 2-8 samples collected after the initiation of ART, were available for analysis. *TRB* repertoires were characterized *via* high-throughput sequencing of the *TRB* variable region performed on genomic DNA extracted from unsorted peripheral blood mononuclear cells. Additional laboratory and clinical metadata including serial measurements of HIV viral load and CD4**^+^** T-cell count were available for all individuals in the cohort. A previously published control group of 189 *TRB* repertoires from peripheral blood samples of adult bone marrow transplant donors was evaluated for comparison. ART initiation in PLHIV was associated with a sustained reduction in viral load and a significant increase in *TRB* repertoire diversity. However, repertoire diversity in PLHIV remained significantly lower than in the control group even after long-term ART. The composition of *TRB* repertoires of PLHIV after ART also remained perturbed compared to the control cohort, as evidenced by large persistent private clonal expansions, reduced efficiency in the generation of TRB CDR3 amino acid sequences, and a narrower range of CDR3 lengths. Network analysis revealed an antigen-experienced structure in the *TRB* repertoire of PLHIV both before and after ART initiation that was quite distinct from the structure of control repertoires, with a slight shift toward a more naïve structure observed after ART initiation. Though we observe significant improvement in *TRB* repertoire diversity with durable viral suppression in PLHIV on long-term ART, the composition and structure of these repertoires remain significantly perturbed compared to the control cohort of adult bone marrow transplant donors.

## Introduction

HIV infection is associated with a profound decrease in the absolute number of circulating CD4**^+^** T-cells that is attributable to a direct viral cytopathic effect as well as indirect “bystander” mechanisms (1). In addition to an absolute loss of T-cell number, HIV infection is associated with oligoclonal expansions in both the CD4**^+^** and CD8**^+^** T-cell compartments (2, 3) and a decrease in T-cell receptor (TCR) diversity (1–3). Long-term treatment with combination antiretroviral therapy (ART) in people living with HIV infection (PLHIV) is associated with suppression of viral replication, durable increases in CD4**^+^** T-cell counts, and improved CD4**^+^** and CD8**^+^** T-cell function (4, 5). Widespread utilization of combination ART over the last two decades has led to a significant improvement in the life expectancy of PLHIV across the globe. However, the life expectancy of PLHIV remains lower than those without HIV infection (6). This is partly attributable to an increased risk of cancer experienced by PLHIV, including both AIDS-defining malignancies as well as other types of cancer such as lung cancer, liver cancer, and Hodgkin lymphoma (7).

Understanding the mechanisms by which long-term ART can lead to reconstitution of the T-cell compartment in PLHIV and improved surveillance against both pathogens and transformed cells, and defining the magnitude of this effect, is a critical research priority. A small number of studies performed before and after the advent of high-throughput DNA sequencing have reported on the impact of chronic HIV infection on the *TRB* repertoire. Analysis of the hypervariable complementarity-determining region 3 (CDR3) region of the T-cell receptor (TCR) in peripheral blood T-cells of PLHIV has revealed increased clonality of the *TRB* repertoire during HIV infection (1–3, 8, 9). A study of CD8**^+^** *TRB* repertoires in 8 participants with untreated, asymptomatic HIV infection observed clonotype-specific changes in the repertoires that corresponded to changes in HIV viral epitope expression (10). These clonotype changes were dependent on viral load, suggesting ongoing evolution of the TCR repertoire in response to HIV epitope changes, which may be critical to maintaining asymptomatic infection (10). PLHIV show a small increase in TCR repertoire diversity after 3 months of ART (2), however, *TRB* repertoires of PLHIV on long-term ART remain significantly perturbed compared to those of control participants, with reduced diversity and prominent idiosyncratic oligoclonal expansions (3).

The effect of long-term ART on the composition and diversity of the peripheral blood *TRB* repertoire has not been evaluated in a serial fashion in individuals. Prior to 2015, when the World Health Organization began recommending immediate initiation of ART in all individuals diagnosed with HIV infection (11), most PLHIV were not started on ART until their CD4**^+^** T-cell counts fell below a certain threshold. In this study, we used serially acquired blood samples and routine clinical and laboratory data from 30 adult PLHIV over a timespan ranging from 2 to 29 years during which ART was initiated in all participants. The earliest samples and data were acquired a decade before the WHO recommendation for immediate ART initiation in PLHIV. High-throughput sequencing of the peripheral blood *TRB* repertoire was performed in each of the 30 adult PLHIV on 1-4 blood samples obtained before ART initiation and 2-8 samples obtained afterward, covering a range of up to 12 years (median, 6 years). The availability of these samples afforded a rare and unique opportunity to evaluate the impact of ART on the diversity and composition of the peripheral blood *TRB* repertoire.

## Materials And Methods

### Study Design

The objective of this retrospective study was to evaluate whether initiation of ART and subsequent durable suppression of HIV replication are associated with changes in the composition and/or diversity of the peripheral blood *TRB* repertoire. The size of the study cohort was determined by the number of participants with confirmed HIV infection from whom at least one peripheral blood mononuclear cell (PBMC) sample collected before ART initiation and at least two PBMC samples collected at different timepoints after ART initiation were available for analysis. The participants included in the study cohort had all been enrolled on clinical studies conducted by the Center for AIDS Research (CFAR) at the University of Washington, Seattle, USA after providing written informed consent.

### Subjects and Samples

A total of 192 cryopreserved PBMC samples serially collected over a mean of 6 (range, 1-12) years from 30 adults with confirmed HIV infection (median, 7 samples per subject) were received from the University of Washington CFAR Biorepository. PBMC samples collected at 1-4 timepoints before and 2-8 time points after the initiation of ART were available from each subject. Additional laboratory and clinical information were obtained for each participant, including HIV viral load, CD4**^+^** T-cell count, antiretroviral therapy history, and other demographic information. MHC genotyping and *TRB* repertoire data for the control cohort was obtained from a previously published study of the *TRB* repertoires of bone marrow transplant donors (12). The median age of the selected control samples was 44 years.

### MHC Genotyping

High-resolution genotyping of the MHC class I and class II loci of all participants in the PLHIV cohort was performed *via* next-generation sequencing as a contract service by Scisco Genetics, Seattle, WA. The full standardized protocol is available online (13). Sequencing of the PCR amplicons spanning the MHC loci was performed on the Illumina MiSeq platform (Illumina, San Diego, CA).

### T-Cell Receptor β-Chain Sequencing

Cryopreserved samples containing 1 – 10 x 10^6^ PBMC were thawed and washed, and genomic DNA was extracted from unsorted PBMC (Qiagen, Hilden, Germany) in batches of 12 using a QIAcube (Qiagen, Hilden, Germany) by the Research Cell Bank at the Fred Hutchinson Cancer Research Center. Prior to extraction of DNA from the study samples, a series of three test extractions were performed on the QIAcube to evaluate the possibility of cross-contamination occurring between samples during processing. Genomic DNA was extracted from a clonal population of T-cells carrying a uniquely rearranged T-cell receptor β-chain (*TRB*) CDR3 nucleotide sequence in parallel with a total of 33 polyclonal T-cell samples, and high-throughput sequencing of the *TRB* variable region was performed on all the individual samples. The unique *TRB* CDR3 sequence carried in the T-cell clone was not observed in any of the 33 polyclonal samples, suggesting that cross-contamination of samples extracted on the QIAcube is negligible. We subsequently extracted genomic DNA from the 192 PBMC samples from the cohort of 30 adult PLHIV on the QIAcube. *TRB* variable region sequencing using the ImmunoSEQ hsTCRB v3.0 assay (Adaptive Biotechnologies, Seattle, WA) was performed on all samples at survey level resolution (14). The final library pool for each set of PBMC samples was sequenced using a v3 150 cycle kit (Illumina, San Diego, CA) on the Illumina MiSeq platform in the FHCRC Genomics Core Facility.

### Repertoire Analysis

Repertoire analyses were conducted using the R statistical programming language (15). A reproducible workflow of all the steps in data QC, data transformation, and analysis was developed using the Nextflow workflow management system (16). Repertoire analysis was performed with open-source R packages (17, 18) and custom scripts were implemented in LymphoSeq2, an R package that we developed for the exploration of Adaptive Immune Receptor Repertoire Sequencing (AIRR-Seq) data (19).

A total of 188 AIRR-Seq repertoires were selected from the 192 genomic DNA samples that were sequenced on the ImmunoSEQ hsTCRB platform. To ensure that all samples considered in the analysis had adequate sequencing coverage, 4 samples from which less than 5000 total *TRB* sequences had been generated were removed from the analysis. A sequence dataset of 189 *TRB* repertoires randomly selected from a previously published dataset of 786 *TRB* repertoires also generated on the ImmunoSEQ platform from peripheral blood samples of bone marrow transplant donors (12) was used as a control population.

To minimize the impact of differences in effective depth of sequencing achieved on the 188 PBMC samples, repertoires were sampled down to a total count of 5000 *TRB* nucleotide sequences. The sampled repertoires were aggregated over the unique *TRB* CDR3 nucleotide sequences and summary statistics were averaged over 100 iterations. Only *TRB* sequences encoding a CDR3 region (defined as the segment starting with the conserved cysteine and ending with the conserved phenylalanine) length of seven or more amino acid residues were retained for the analysis. The total count of sequences detected in a repertoire is calculated as the sum of the counts for each *TRB* CDR3 sequence identified in the repertoire. Unique productive sequences are calculated as the total number of unique *TRB* sequences that generate productive CDR3 amino acid sequences. TCR sequence production efficiency is calculated as the average number of *TRB* nucleotide sequences that encode the same TRB CDR3 amino acid sequence in each repertoire. Diversity of the encoded CDR3 amino acid sequences from the *TRB* repertoire was estimated using the clonality score, a derivative of the Shannon-Weaver entropy index, and the Gini coefficient.

TRB CDR3 amino acid length distributions were calculated by counting the number of unique CDR3 amino acid sequences of a particular length found in the entire repertoire. Pairwise similarity score between two repertoires was calculated as the ratio of total number of unique productive TRB CDR3 amino acid sequences shared by the repertoires to the total number of sequences in both repertoires. To build a TCR network for each repertoire, we sampled the most frequent 5000 CDR3 amino acid sequences from each repertoire and connected CDR3 amino acid sequences (nodes) that differed by an edit distance of 1 by an edge. A degree distribution curve was generated by calculating the cumulative frequency of nodes of a given degree in the repertoires of the three sample groups (pre-ART, post-ART, control). A regression curve was generated for each group using a Generalized Additive Model (GAM) to represent the trend in degree distribution (number of connections) across the three groups. All visualizations were built using ggplot2 (20), ggpubr (21) was used to generate boxplots, ggalluvial (22) was used to generate alluvial diagrams, and ggraph (23) and tidygraph (24) were used to visualize a TCR network for each repertoire.

### Prediction of Antigenic Specificity and MHC Restriction

Antigenic specificity and MHC-restriction of TRB CDR3 amino acid sequences were computationally predicted using the publicly available GLIPH2 algorithm (25). Sequences from repertoires were labeled with repertoire ID and phenotype (PLHIV, control). MHC typing information for the control cohort was obtained from the immunoSEQ Analyzer portal (Adaptive Biotechnologies, Seattle, WA; https://clients.adaptivebiotech.com/pub/Dean-2015-GenomeMed) when available. MHC typing information from both the PLHIV and control cohorts was formatted as per GLIPH2 requirements.

The TRB CDR3 amino acid sequences in each repertoire were compared with the VDJdb and McPAS-TCR (26–28) databases of annotated TRB sequences to identify sequences that have previously been associated with a T-cell response to a specific microbial or tissue antigen or a specific pathological condition. TRB CDR3 amino acid sequences with likely antigenic specificity for CMV, EBV, HIV, or an identified tissue antigen, were identified by first identifying all specificity groups that contained an annotated TRB sequence reported in the one or both databases. These specificity groups were further filtered by the MHC restriction predicted by GLIPH2 to generate predicted antigen specific GLIPH2 groups with specific MHC restriction. All TRB CDR3 amino acid sequences belonging to these specificity groups were identified as sequences with likely antigenic specificity against CMV, EBV, HIV, a specific tissue antigen, or pathological condition.

### Statistical Analysis

Comparison of means between the pre-ART, post-ART, and control repertoires was performed with the Wilcoxon signed-rank test. To correct for multiple testing, we used the Holm-Bonferroni correction. An adjusted p-value of <0.05 was considered significant.

### Study Approval

All samples and clinical and laboratory data analyzed in this study were from participants who had given written informed consent using forms approved by the Center for AIDS Research at the University of Washington (Seattle, Washington, USA) or the Fred Hutchinson Cancer Research Center IRB (Seattle, Washington, USA).

## Results

The study cohort consists of 30 adults with confirmed HIV infection from whom 192 peripheral blood samples were collected over the span of 2-12 years (**Table 1**), representing a comprehensive cohort of serially collected pre- and post-ART samples from individuals with HIV. All participants were treated in the United States of America. Eleven out of 30 participants in the HIV cohort were female. The median age at the time of the first PBMC sample was 39 years. Serial viral load and CD4^+^ T-cell counts from the time of enrollment were also obtained for each of the participants. Sequencing of the T-cell receptor β-chain repertoire from each of the 192 PBMC samples was performed at a targeted depth of 40,000 – 50,000 T-cell genomes per sample. Along with the PLHIV repertoires, 189 *TRB* repertoires from the PBMCs of 189 bone marrow transplant donors were obtained from a previously published dataset to serve as a control population (**Table 1**).

**Table 1:** Characteristics of the PLHIV and control cohorts and sample sets, with mean and range of the total number of unique productive TRB CDR3 amino acid sequences and total number of *TRB* nucleotide sequences in the repertoires in each cohort.

Category	PLHIV	Control
Number of individuals	30	189
Number of blood samples	192	189
Number of repertoires with >5000 *TRB* nucleotide sequences	188	189
Median time on ART	9 years (2-13)	NA
No. of Pre-ART repertoires	59	NA
No. of Post-ART repertoires	129	NA
No. of Normal repertoires	NA	189
Total number of unique productive TRB CDR3 amino acid sequences	27,504 (5,952-64,598)	223,120 (35,415-477,806)
Total count of sequences	50,746 (7,830-90,148)	4,226,814 (560,260-26,798,209)

NA, not applicable.

### Durable Viral Suppression With Long-Term ART Is Weakly Correlated With an Increase in CD4^+^ T-Cell Count and Repertoire Diversity

**Figure 1:**
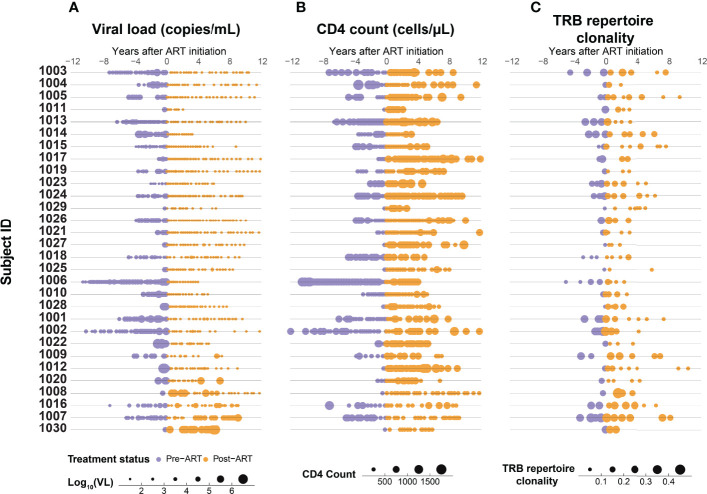
Suppression of HIV replication with ART is associated with a gradual increase in CD4^+^ T-cell count and decrease in T-cell β-chain repertoire clonality. Serial measurements of **(A)** HIV viral load, **(B)** CD4^+^ T-cell count, and **(C)** clonality of the peripheral blood *TRB* repertoire in 30 adults with HIV infection determined up to 12 years before (purple) and after (orange) ART initiation.

**Figure 2:**
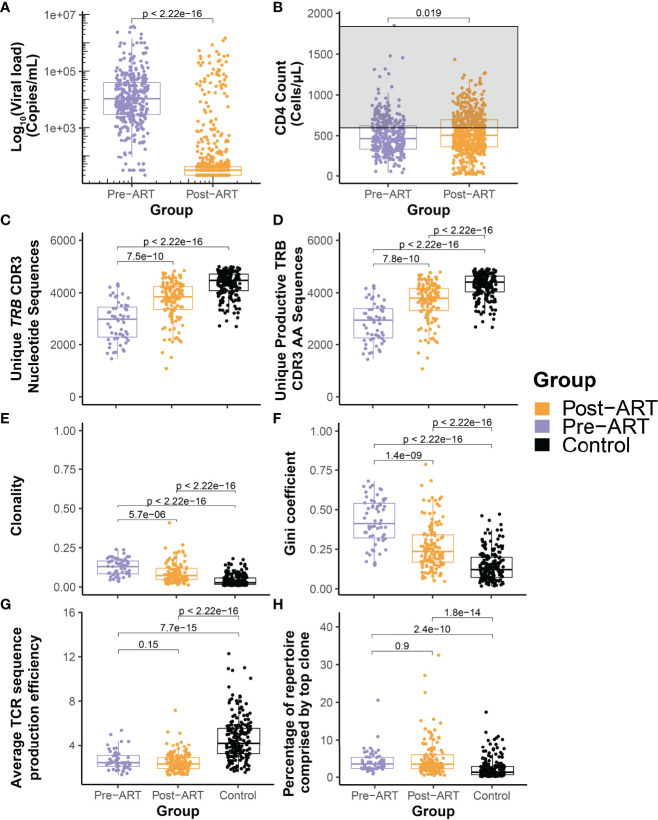
Durable suppression of HIV with ART is characterized by significant diversification of the T-cell β-chain repertoire but persistence of large clonal expansions and perturbed architecture. Pooled distributions of all log_10_ HIV viral load **(A)** and CD4^+^ T-cell count **(B)** measurements obtained from the cohort of 30 PLHIV up to 12 years before (purple) and after (orange) initiation of ART. The grey region in **(B)** indicates the generally accepted normal range of the peripheral blood CD4^+^ T-cell count. **(C)** Total number of unique *TRB* CDR3 nucleotide sequences and **(D)** number of unique productive TRB CDR3 amino acid sequences in 59 *TRB* repertoires assessed before (purple) and 129 repertoires assessed after (orange) initiation of ART in the cohort of 30 PLHIV, and from 189 control bone marrow transplant donors each evaluated at a single time point. **(E)** Clonality of the pre- and post-ART PLHIV repertoires and of the control repertoires. **(F)** Equality of the TRB CDR3 amino acid sequence distributions in the pre-ART, post-ART, and control repertoires as captured by the Gini coefficient. **(G)** Mean TRB sequence production efficiency. **(H)** Percentage of total repertoire sequence count contributed by the most frequent TRB CDR3 amino acid sequence.

Serial HIV viral load measurements and CD4^+^ T-cell counts were collected from the 30 PLHIV over intervals ranging from 2-23 years that began prior to ART initiation and in almost all cases spanned at least the first two years after ART (**Figure 1**). The initiation of ART is associated in almost all participants with an immediate reduction in viral load, followed by durable viral suppression with long term ART (**Figure 2A**). While a steady increase in CD4^+^ T-cell count is observed in most PLHIV with continued ART, there is only a weak correlation between viral suppression and restoration of CD4^+^ T-cell counts (**Figure 2B**). We observe that even after 10 or more years of ART, the CD4 counts in this cohort of PLHIV generally did not return to the range observed in seronegative individuals (700 – 1800 cells/μL).

To understand the change in *TRB* repertoire characteristics, we compared the depth normalized summary metrics of the PLHIV repertoires with those from a control cohort comprised of allogeneic bone marrow transplant donors. With the initiation of ART, we see a significant improvement in repertoire characteristics of PLHIV with an increase in the total number of unique *TRB* CDR3 nucleotide sequences and unique productive TRB CDR3 AA sequences found in the repertoire (**Figures 2C, D**). There is a significant decrease in the clonality and Gini coefficient, indicating an increase in repertoire diversity (**Figures 2E, F**). However, when compared to the control cohort of bone marrow transplant donors, we can see that the repertoire size and diversity remains perturbed in PLHIV despite long-term ART. Within the time frame of observation in this study, we see that despite durable viral suppression there is limited restoration of the *TRB* repertoire in PLHIV on ART. Evaluation of the number of unique CDR3 nucleotide sequences that encode a single CDR3 amino acid sequence demonstrates that repertoires of PLHIV before and after ART have significantly lower average TRB sequence production efficiency than those of the control cohort (**Figure 2G**), demonstrating in a different dimension the reduced diversity in PLHIV repertoires.

### T-Cell β-Chain Repertoires of PLHIV Are Dominated by Large Clonal Expansions That Pre-Date ART Initiation and Persist for Years Despite Therapy

A population of relatively expanded *TRB* sequences that appears early within the first several years of HIV infection and persists through and beyond the initiation of ART dominates the *TRB* repertoire in PLHIV. Indeed, many PLHIV repertoires are characterized by a single particularly abundant sequence that appears prior to treatment and persists long after ART initiation. The most abundant *TRB* sequence in both pre- and post-ART repertoires from PLHIV comprises a significantly larger fraction of the repertoire than is seen in control group repertoires (**Figure 2H**).

**Figure 3:**
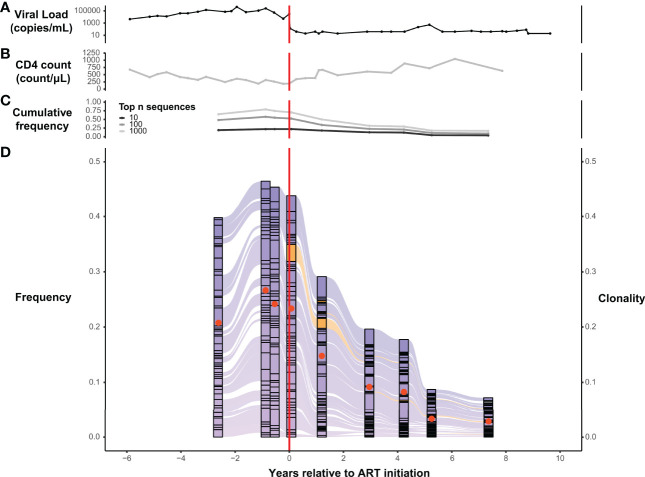
T-cell β-chain repertoires from PLHIV are dominated by large clonal expansions that can persist for years following initiation of ART. Serial measurements obtained over a span of 14 years, before and after ART initiation, of HIV viral load **(A)** and CD4^+^ T-cell count **(B)** in a representative participant, 1001, from the PLHIV cohort. **(C)** Cumulative frequency of the most frequent 10, 100, and 1000 TRB CDR3 amino acid sequences in the blood *TRB* repertoire of participant 1001 measured at 9 different timepoints over a 10-year interval spanning the initiation of ART. **(D)** Alluvial plot of the 9 different *TRB* repertoires from participant 1001 showing the variation in frequency (left axis) of the 50 most frequent TRB CDR3 amino acid sequences in the blood. Sequences first observed before ART initiation are colored purple and those first observed after ART initiation are colored orange. The clonality (right axis) of each repertoire is represented as a filled (tangerine) circle superimposed on the corresponding component of the alluvial plot. Dashed vertical red line indicates the timing of ART initiation.

Serial analysis of specific TRB sequences in the repertoires of individual participants reveal the surprising durability of expanded T-cell clones. For example, tracking the most abundant 50 TRB amino acid sequences in the repertoires of representative participant 1001 across time, we see that the dominant sequences that persist across more than 10 years of serial observation were significantly expanded in the peripheral blood before the initiation of ART (**Figure 3D**). During this 10+ year interval, which spanned the initiation of ART, the cumulative frequency of the most abundant 10, 100, and 1000 TRB sequences in participants 1001 gradually declined while the CD4^+^ T-cell count gradually increased (**Figures 3B, C**), suggesting that CD4**^+^** T-cells may play a role in regulating the prominent clonal expansions seen in PLHIV repertoires. The gradual improvement of the CD4**^+^** T-cell count seen over several years after ART initiation contrasts sharply with the dramatic drop in HIV viral load that occurred immediately after therapy was started (**Figure 3A**).

**Figure 4:**
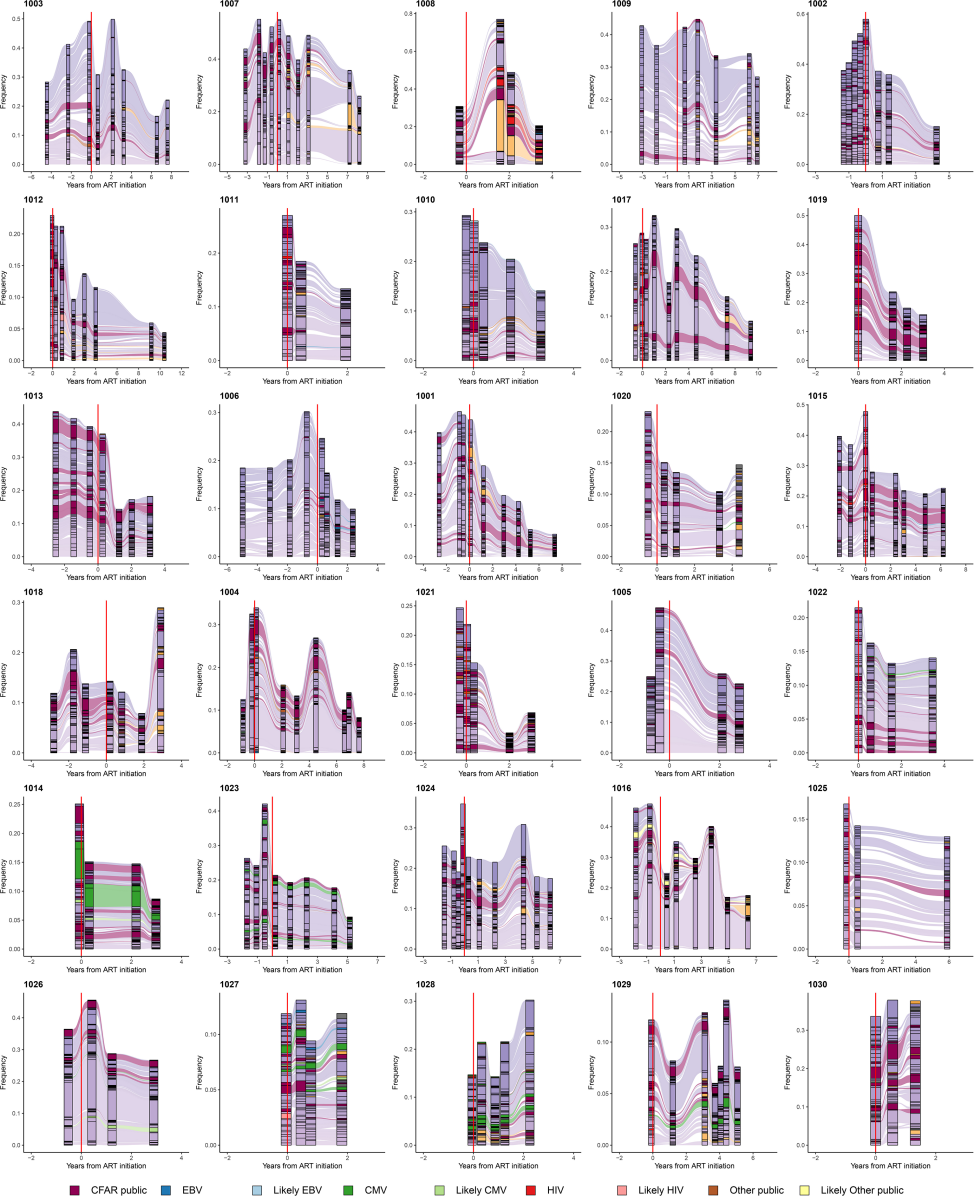
Persistently expanded TRB CDR3 amino acid sequences in PLHIV repertoires comprise primarily private T-cell responses. Alluvial plots demonstrating the variation in frequency over time of the most frequent 50 TRB CDR3 amino acid sequences in the repertoires of 30 PLHIV. TRB CDR3 amino acid sequences described in VDJdb (26, 27) or the McPAS-TCR database (28) that have been associated with T-cell responses against specific viral antigens encoded by EBV (blue), CMV (green), or HIV (red), or other sources (brown), are colored accordingly. TRB CDR3 amino acid sequences predicted by GLIPH2 (25) to share similar or identical antigenic specificity with these annotated sequences are colored light blue, light green, light red, or light brown, respectively. Annotated sequences are only highlighted if the participant in whom the sequences were observed was found by MHC genotyping to express the specific MHC class I allele associated with the relevant sequence in the database. TRB CDR3 amino acid sequences observed in the repertoire of two or more PLHIV but not described in the two annotated databases are colored maroon. All other sequences are colored purple if they were first observed pre-ART initiation or orange if they were first observed post-ART. The dashed vertical red lines indicate the timing of ART initiation for each PLHIV.

We similarly tracked the 50 most abundant TRB sequences across time within each set of PLHIV repertoires and observed that expanded TRB clones identified before the initiation of ART dominate the repertoire across all time points. The magnitude of that dominance fluctuates over time and decreases in many PLHIV after ART (**Figure 3D**; **Figure 4**). Although abundant TRB sequences that were not expanded in any pre-ART repertoire sometimes appeared to be expanded in post-ART repertoires (orange sequences in **Figures 3D** and **Figures 4**), such sequences were uncommon. Thus, most of the clonal expansions seen in the repertoires of PLHIV on ART are not due to *de novo* T-cell responses elicited in some manner by ART initiation.

### Persistently Expanded β-Chain Clones in the T-Cell Repertoires of PLHIV Are Largely Private

We systematically interrogated VDJdb (26, 27) and the McPAS-TCR database (28) of annotated TRB sequences to determine whether TRB sequences carried in the 188 PLHIV and 189 control repertoires had previously been associated with a T-cell response to a specific microbial or tissue antigen or a specific pathological condition. Included in the sequences annotated in VDJdb and the McPAS-TCR database are many “public” TRB sequences that (i) have been shown to be carried in T-cells specific for canonical peptide antigens derived from pathogens such as CMV, EBV, and HIV, and presented by specific MHC alleles, and (ii) identified in multiple individuals who express the relevant MHC allele. Most of the expanded TRB CDR3 amino acid sequences present in the repertoires of PLHIV were neither identified in VDJdb or the McPAS-TCR database, nor observed at any frequency in the repertoires of other PLHIV in the cohort. Thus, the clonally expanded T cell populations that dominate the repertoires of PLHIV before and after ART are, with some exceptions described below, largely private to each individual, and their antigenic specificity is unknown.

A small minority of the TRB sequences in the PLHIV repertoires were identified in either VDJdB or the McPAS-TCR database, including sequences associated with well-characterized public T-cell responses to CMV, EBV, and HIV. The availability of MHC genotyping data for the PLHIV cohort allowed us to confirm that PLHIV in whom such public sequences were identified almost always carried the specific MHC allele associated with each sequence. For example, two similar TRB CDR3 sequences, CASSIVTGPYNEQFF and CASSIVQGPYNEQFF, encoded in part by the *TRBV19* and *TRBJ2-1* gene segments and associated with a CD8**^+^** T-cell response to the CMV IE2-derived peptide NEGVKAAW presented by HLA-B*44 (26, 27, 29, 30), were detected at high frequency in all 4 repertoires obtained over 3 years from participant 1014 (**Figure 4**), whose MHC genotype included HLA-B*44:03:01.

We used the GLIPH2 algorithm (25) to identify within each repertoire “specificity groups” of TRB sequences with predicted similar or identical antigenic specificity and MHC restriction. This analysis predicted, for example, that the TRB sequences CASSIVTGPYNEQFF and CASSIVQGPYNEQFF observed at high frequency in all of the repertoires from participant 1014 and associated with CD8**^+^** T-cells specific for NEGVKAAW presented by HLA-B*44 belonged to the same TRB specificity group. The identification of such specificity groups allowed us to leverage the annotation available in VDJdb and McPAS-TCR to identify additional TRB sequences in PLHIV repertoires that heretofore were not known to be associated with a T-cell response to a specific antigen but are predicted to share antigenic specificity with previously described CMV-, EBV-, HIV-, or tissue-specific T-cell responses (identified as “Likely CMV”, “Likely EBV”, “Likely HIV” or “Likely Other public” sequences in **Figure 4**).

We compared the frequency with which annotated sequences described in VDJdb or the McPAS-TCR database were observed in the pre-ART, post-ART, and control repertoires. We found no significant overabundance of previously characterized CMV, EBV-, or HIV-associated sequences in the PLHIV compared with the control repertoires (**Supplementary Figure 1**). In fact, annotated sequences described in the two databases, disregarding their reported MHC association and the MHC genotype of the participant in which they were observed, were significantly more abundant in the control repertoires as compared to those from PLHIV.

### Candidate Public TRB Sequences of Unknown Antigenic Specificity

Although PLHIV repertoires were largely private to each individual, we did identify a small number of TRB sequences that were observed in multiple repertoires from two or more PLHIV but are not listed in VDJdB or the McPAS-TCR database. Moreover, the MHC genotypes of the participants in whom these sequences were observed in many cases shared one or more class I or class II alleles, suggesting the possibility of public MHC-restricted responses against as yet unidentified antigens. The TRB CDR3 sequence CAWSVLKGETQYF, for example, is not described in VDJdb or the McPAS-TCR database, but it was observed in 5/5 repertoires from participant 1010, 7/9 repertoires from participant 1017, and 8/8 repertoires from participant 1023. It was not observed in repertoires from any of the 27 other PLHIV. The MHC genotypes of participants 1010, 1017, and 1023 share HLA-B*44:02:01, suggesting that CAWSVLKGETQYF may be carried in a public T-cell receptor against an as yet unidentified antigen presented by HLA-B*44:02.

### Similarity Between PLHIV Repertoires Is Significantly Lower Than That Between Control Repertoires

**Figure 5:**
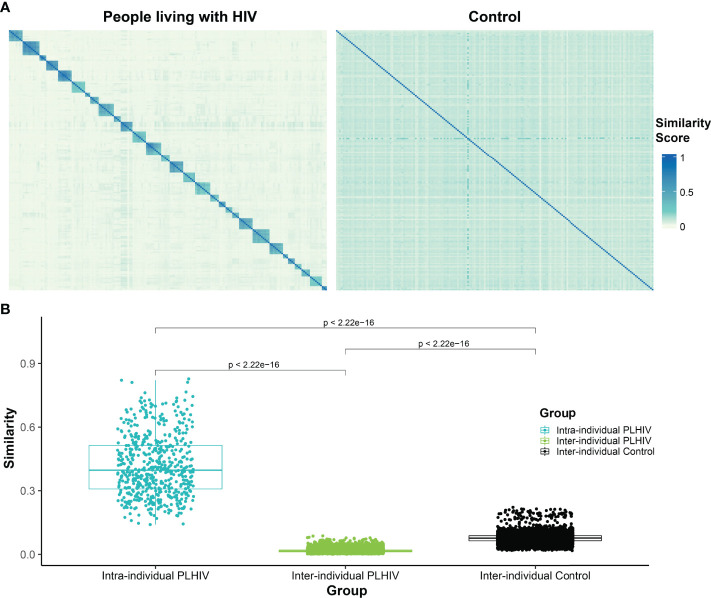
T-cell β-chain repertoires of PLHIV demonstrate significantly less inter-individual similarity than control repertoires. **(A)** Heatmap of pairwise similarity scores between the repertoires of 30 PLHIV (left) and between 189 control bone marrow transplant donors (right). In the PLHIV cohort, repertoires are ordered by participant ID followed by the date of sample collection. **(B)** Boxplot demonstrating the distribution of pairwise similarity scores within-participants and across- participants in the PLHIV cohort and in the control cohort.

To further evaluate the apparent dominance of private T-cell responses in PLHIV repertoires, we measured the pairwise repertoire similarity scores between repertoires derived from the same participant, as well as the similarity scores between repertoires derived from different participants, in the PLHIV cohort. We then compared these against the pairwise similarity scores between the single repertoires from each of the 189 individuals in the control group (**Figures 5A, B**). As expected, the intra-individual similarity scores were significantly higher than the inter-individual similarity scores in PLHIV repertoires (**Figure 5B**). Moreover, the inter-individual similarity scores in PLHIV were significantly lower than the inter-individual similarity scores in the control group (**Figure 5B**). Given that 129 of the 188 PLHIV repertoires included in this analysis were obtained from individuals on ART, this demonstrates that PLHIV repertoires remain markedly private and idiosyncratic, as has been previously observed (3), despite long-term ART.

### TRB CDR3 Length Distributions Exhibit a Narrower Range in PLHIV

**Figure 6:**
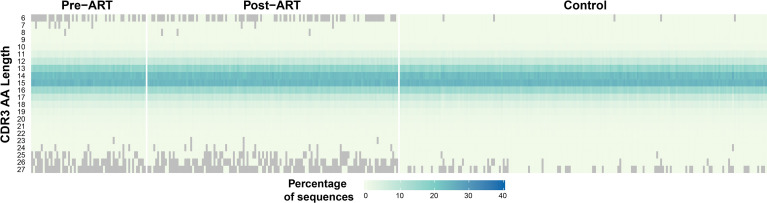
Narrower distribution of CDR3 region lengths encoded in *TRB* sequences in PLHIV repertoires compared to control repertoires. ​​Heatmap demonstrating the length distribution (plotted along vertical axis) of the CDR3 regions encoded by the *TRB* sequences in the pre- and post-ART repertoires of the PLHIV cohort (left side of figure) and in 189 bone marrow transplant donors (right side of figure) each sampled at a single timepoint.

The median length of the CDR3 region in the T-cell receptor β-chain in repertoires from healthy adults is 15 AA (31). We examined the distribution of TRB CDR3 lengths in the pre-ART, post-ART, and control *TRB* repertoires (**Figure 6**). The median CDR3 AA sequence length in the pre-ART and control group repertoires was 15 AA, but 14 AA in post-ART repertoires. Of greater potential significance is the observation that the range of CDR3 length distributions in both pre- and post-ART PLHIV repertoires is narrower than that observed in the control group. In contrast to the control repertoires, many pre- and post-ART repertoires contain few, if any, short TRB CDR3 sequences of length 6 AA, and few, if any, long CDR3 sequences of length 25, 26, or 27 AA. The finding of a narrower distribution of CDR3 sequence length in *TRB* repertoires in PLHIV on long-term ART when compared with control repertoires is at odds with observations reported in a previous study (3). However, our findings are consistent with those of prior studies that have shown an enrichment of TRB CDR3 sequences of shorter lengths in antigen-experienced repertoires (32, 33).

### T-Cell β-Chain Repertoire Structure Shifts Toward a Naive Repertoire Structure With Long-Term ART

**Figure 7:**
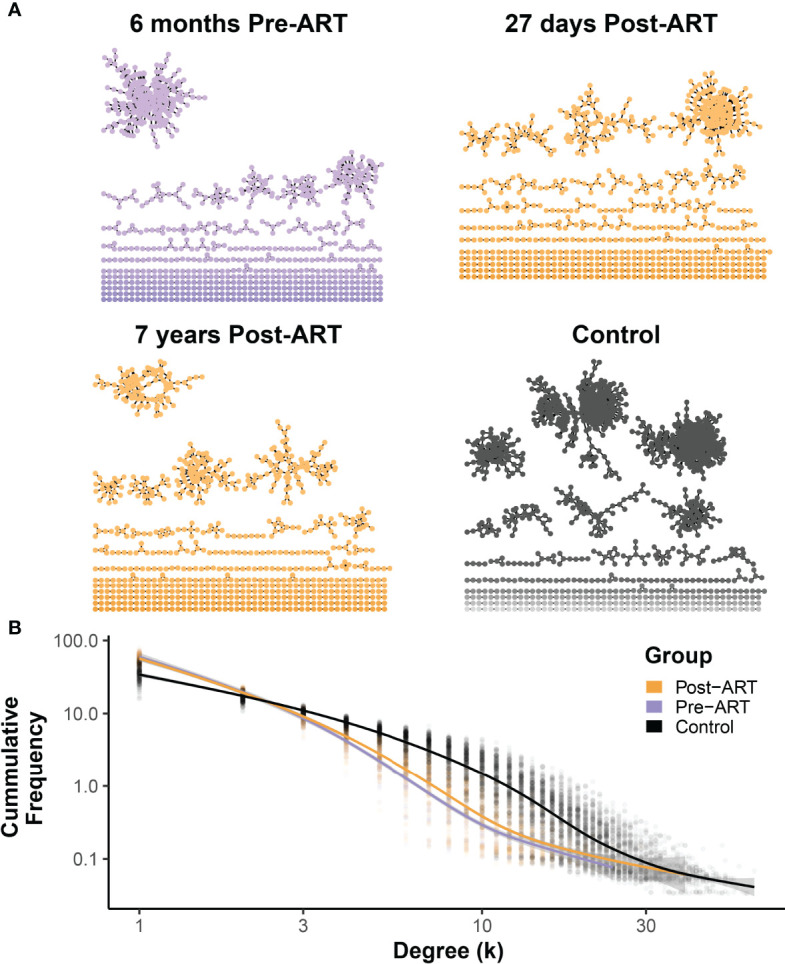
Network structures of pre-ART T-cell β-chain repertoires in PLHIV resemble antigen-experienced repertoires dominated by one-to-many connections, with a small shift following ART initiation toward a many-to-many structure observed in control repertoires. **(A)** Representative TCR networks of the most frequent 5000 TRB sequences from in the *TRB* repertoire of PLHIV participant 1001 evaluated at 6 months before ART, 27 days after initiation ART, and 7 years after initiation of ART, and the repertoire of control participant HIP09062. TCR networks were constructed for each of the 188 PLHIV repertoires and the 189 control repertoires by connecting the CDR3 amino acid sequences that differed by an edit distance of 1 with an edge. Each unique CDR3 amino acid sequence forms a node in the network. **(B)** The degree (number of connections) of each CDR3 amino acid sequence (node) in the network was calculated for the each of 188 PLHIV repertoires and the 189 control repertoires, and the frequency distribution of nodes of different degrees (k) of connectedness for the pre- (purple) and post-ART (orange) PLHIV repertoires and for the 189 control repertoires is plotted. The thick lines represent GAM regression lines tracking the frequency of nodes by degree for each group.

To evaluate the impact of ART on the architecture of the *TRB* repertoire in PLHIV, we generated a network visualization of each pre-ART, post-ART, and control repertoire by connecting TRB CDR3 sequences that differed by 1 AA with an edge (representative data from participant 1001 in **Figure 7A**). We calculated the degree of each node in the network and plotted the degree distribution for all the repertoires in each of the three groups (pre-ART, post-ART, control). Previous studies have shown that naïve *TRB* repertoires exhibit an exponential degree distribution curve dominated by many-to-many interactions in the T-cell network (34, 35). This has been attributed to the increased diversity typically observed in naïve repertoires, which results in a higher likelihood of finding sequences with an edit distance of one. In contrast, repertoires with a larger antigen-experienced component demonstrate a power-law degree distribution curve dominated by one-to-many network interactions. Looking at the degree distribution curves across the three groups, we can see that the control repertoires demonstrate degree distribution curves that are exponential in nature. The degree distribution curves of the pre- and post-ART PLHIV repertoires, in contrast, demonstrate characteristics that are less exponential and more power-law in nature (**Figure 7B**). Comparison of the degree distribution curves for the pre- and post-ART repertoires reveals that the post-ART repertoires have a slightly larger number of high degree (≥3) nodes than the pre-ART repertoires. This difference implies a higher degree of repertoire diversity in the post- compared with the pre-ART repertoires. Although the magnitude of the difference between the degree distribution curves is small, it is consistent with the notion that post-ART repertoires have increased diversity, which in turn suggests a greater naïve component.

## Discussion

The introduction of combination ART for the treatment for HIV infection has led to a marked reduction in the number of AIDS-related deaths and significantly longer life expectancy in PLHIV (36). These advances are in large part attributable to the increase in CD4**^+^** T-cell counts associated with effective suppression of HIV replication (37). However, PLHIV on long-term combination ART still experience significantly shorter life expectancy when compared to age-matched seronegative individuals (6, 7, 38). The reduced life expectancy of PLHIV despite highly active combination ART is multifactorial, but increased mortality due to cancer is in part responsible (38–41). Identifying the factors that underlie this increased cancer mortality is a high priority.

Research over many decades has demonstrated the critically important role of the immune system in preventing and treating cancer. T-lymphocytes in particular have become powerful weapons in the therapeutic arsenal, and increasing evidence demonstrates that they also play an important role in immune surveillance against cancer (42–44). This study is motivated by the recognition that persistent T-lymphocyte immune deficiency in PLHIV on long-term ART could limit or impair effective immune surveillance against transformed cells, yet our knowledge of the impact of long-term ART on the *TRB* repertoire is quite incomplete.

In this study we utilized a unique sample and data set to perform the first comprehensive serial analysis of the peripheral blood *TRB* repertoire in PLHIV treated with long-term combination ART. The availability of these resources allowed us to follow with unprecedented detail and precision the impact of ART initiation on the reconstitution of the *TRB* repertoire in 30 PLHIV over a median of 6 years. We established that the *TRB* repertoire in PLHIV remains decidedly perturbed, and likely impaired, despite long-term treatment with combination ART. Although gradual improvement in the CD4**^+^** T-cell counts of the 30 PLHIV in our study cohort over the period of observation was observed, many did not recover a count within the normal range (>700/μL), confirming recent studies by other groups (45). When compared with the *TRB* repertoires of 189 healthy bone marrow transplant donors evaluated with the same wet- and dry-lab platforms, the repertoires of the 30 PLHIV remained decidedly perturbed despite long-term ART and effective viral suppression in most participants. PLHIV repertoires on long-term ART remained significantly more clonal and less diverse than control repertoires, with persistent oligoclonal expansions of *TRB* sequences that, with few exceptions, developed before ART initiation. They display significantly reduced TRB sequence production efficiency, a narrower range of CDR3 lengths – particularly very long CDR3 sequences, reduced similarly to each other and to control repertoires, and network architecture that also reflects less diversity. While we hypothesize that these persistent oligoclonal expansions reside primarily within the CD8**^+^** T-cell compartment, a number of previously published studies have described the existence of persistent oligoclonal expansions of CD4**^+^** T-cells that carry HIV proviruses integrated at specific sites within the genome in PLHIV on long-term ART (46–48).

A reduction in T-cell diversity is commonly seen in aging HIV-seronegative individuals (49, 50). The 30 PLHIV in our study were between 23 and 54 years old at the time of their earliest PBMC sample. The median age of the 189 bone marrow donors that comprised the control group was 41 years. Thus, we do not feel that age *per se* can explain the profound perturbation observed in the PLHIV repertoires.

This study has several important limitations. The blood samples from PLHIV were obtained between 2004 and 2017, and the participants were treated with combination ART regimens that may not be as effective as those that are currently available. Critical information regarding adherence to ART, viral resistance, and changes in therapy for the PLHIV was not available, limiting the scope of the inferences that could be made regarding the changes in the *TRB* repertoire after initiation of ART. The period of observation, although extending over several years for each of the 30 participants in the PLHIV cohort, could have been longer. We examined the repertoire of unsorted T-cells, rather than sorted CD4**^+^** and CD8**^+^** T-cells, due to the limited number of PBMC available from most of the participants prior to ART initiation, when they were uniformly profoundly lymphopenic. The α and β-chains of the T-cell receptor collectively determine antigenic specificity. However, due to limited number of PBMC available for many of the time points, in the current study we were only able to study the T-cell receptor β-chain (51, 52). Nonetheless, we feel that the observations made in this study are well founded and provide a useful framework within which to direct and pursue future studies.

Our results raise a number of important questions. Network analysis has previously been used to show the differences in repertoire structure of naïve and antigen-experienced repertoires (35, 53, 54), and we find that the network structure of PLHIV repertoires shows an antigen-experienced characteristic with a predominantly one-to-many structure. However, the network structure of the post-ART repertoires shows a slight shift towards the many-to-many structure observed in the control cohort. We hypothesize that this shift post-ART is due to the recovery of the naïve T-cell compartment with initiation of ART. Prior studies in adolescents with HIV have shown that perturbations in the naïve CD4**^+^** T-cell compartment steadily decline in magnitude after the initiation of ART, and these repertoires become increasingly similar to those seen in seronegative adolescents (55). Future studies are needed to evaluate the effects of ART on the naïve and memory T-cell compartments in adults with HIV. However, our observation of a slight shift in degree distribution with long-term ART suggests that initiation of ART at the time of diagnosis, which is the current standard of care, could lead to superior immune restoration in PLHIV. Studies of PLHIV who were promptly started on combination ART at the time of diagnosis could answer this question. A published study (3) of 26 PLHIV who had been on combination ART for a median of 8.5 years likewise demonstrated persistently perturbed *TRB* repertoires with significantly reduced diversity, but whether these participants had initiated ART immediately after diagnosis was not stated. Our observations and those of others (3) highlight the importance of identifying the phenotypic characteristics of the T-cell repertoire and whether ART initiation is associated with changes in the phenotype. Future studies could also utilize TREC analysis (56) to determine whether long-term ART is associated with improvement in naïve T-cell production in PLHIV.

Determining whether the perturbed *TRB* repertoires demonstrated in this as well as previous (3) studies could be pathophysiologically linked to the increased risk of cancer morbidity and mortality faced by PLHIV will require considerable additional study. The malignancies for which PLHIV are at elevated risk include virus-associated cancers such as cervical carcinoma and other HPV-related cancers, hepatocellular carcinoma, and Kaposi sarcoma, but also include diseases such as lung cancer that are not thought to be pathogen-associated. Several mechanisms of increased susceptibility may be at play and may contribute differentially to the risk of specific cancers. There were an estimated 37.7 million PLHIV across the globe at the end of 2020 (57), over two thirds of whom are in the WHO African Region, where resources for cancer prevention, diagnosis, and care are more limited than in high income countries. Thus, further studies of the *TRB* repertoire in PLHIV and its influence on the risk for cancer development are urgently required.

## Data Availability Statement

TRB repertoire sequencing data from the 192 peripheral blood samples is publicly available for access on the Adaptive immunoSEQ Analyzer portal (https://clients.adaptivebiotech.com/pub/towlerton-2022-hiv). The control dataset was sampled from the 786 TRB repertoire sequencing datasets from bone-marrow donors available on the Adaptive immunoSEQ Analyzer portal (Adaptive Biotechnologies, Seattle, WA; https://clients.adaptivebiotech.com/pub/Dean-2015-GenomeMed). All R scripts and Nextflow workflows to analyze and visualize the data used in the study is available on GitHub (https://github.com/shashidhar22/airr_seq_pipelines).

## Ethics Statement

The studies involving human participants were reviewed and approved by the Center for AIDS Research at the University of Washington (Seattle, Washington, USA) or the Fred Hutchinson Cancer Research Center IRB (Seattle, Washington, USA). The patients/participants provided their written informed consent to participate in this study.

## Author Contributions

EW and AT conceived and designed the study. AT generated the *TRB* sequence data. SR, DC, AT, and EW analyzed the data. SR, AT, and EW drafted the manuscript. All authors edited the manuscript. All authors contributed to the article and approved the submitted version.

## Funding

This research was supported by an award from the National Cancer Institute (U54 CA190146) and by the University of Washington Center for AIDS Research (CFAR), an NIH funded program (P30 AI027757) which is supported by the following NIH Institutes and Centers: NIAID, NCI, NICHD, NHLBI, NIDA, NIMH, NIA, NIGMS, NIDDK, FIC, and OAR. This research was also supported by the Specimen Processing and Research Cell Bank and the Genomics and Bioinformatics Shared Resources of the Fred Hutch/University of Washington Cancer Consortium (P30 CA015704). Bioinformatic analysis was supported by the Scientific Computing Infrastructure at Fred Hutch funded by ORIP grant S10OD028685.

## Conflict of Interest

The authors declare that the research was conducted in the absence of any commercial or financial relationships that could be construed as a potential conflict of interest.

## Publisher’s Note

All claims expressed in this article are solely those of the authors and do not necessarily represent those of their affiliated organizations, or those of the publisher, the editors and the reviewers. Any product that may be evaluated in this article, or claim that may be made by its manufacturer, is not guaranteed or endorsed by the publisher.

## Supplementary Material

Supplementary File 2 CFAR_Dean_metadata.xlsx: Clinical and sequencing metadata for the PLHIV and Control cohort.
