# Supplementary material for: Serial Analysis of the T-Cell Receptor β-Chain Repertoire in People Living With HIV Reveals Incomplete Recovery After Long-Term Antiretroviral Therapy
Source: Front Immunol. 2022 May 2;13:879190. doi: 10.3389/fimmu.2022.879190 (PMC9108698; doi:10.3389/fimmu.2022.879190)
Supplement: Supplementary file 1 [file DataSheet_1.docx]

Supplementary Material

# Supplementary Figures

**
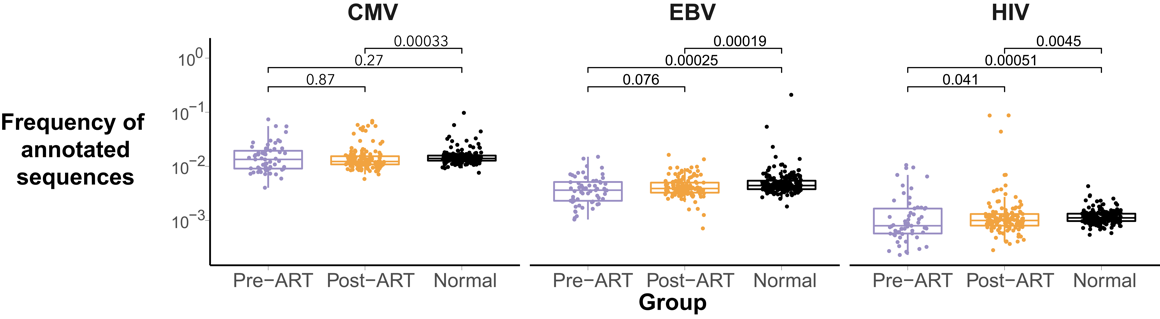
**

**Supplementary Figure 1.** ***TRB* sequences found in annotated databases comprise a small minority of sequences in PLHIV T-cell repertoires.** Frequency distribution of *TRB* sequences in the pre- (purple) and post-ART (orange) repertoires of 30 PLHIV and in the control population of bone marrow transplant donors (black) that are described in two curated databases, VDJdb (26,27) or the McPAS-TCR database (28).
